# Immune checkpoint inhibitor-related acquired amegakaryocytosis thrombocytopenia: a case report and literature review

**DOI:** 10.3389/fonc.2024.1353896

**Published:** 2024-03-07

**Authors:** Valérian Rivet, Vincent Sibaud, Jérémie Dion, Thibaut Volosov, Mélanie Biteau, Andréa Pastissier, Karen Delavigne, Pierre Cougoul, Odile Rauzy, Thibault Comont

**Affiliations:** ^1^ Internal Medicine and Immunopthology Department, The Cancer University of Toulouse Oncopole, University Hospital Center of Toulouse, Toulouse, France; ^2^ Dermatology Department – Medical Oncology, The Cancer University of Toulouse Oncopole, University Hospital Center of Toulouse, Toulouse, France; ^3^ The Anatomical Pathology Department, The Cancer University of Toulouse Oncopole, University Hospital Center of Toulouse, Toulouse, France

**Keywords:** acquired megakaryocytic thrombocytopenia, immune checkpoint inhibitors (ICI), immune-related adverse events (IRAE), nivolumab, eltrombopag, thrombopoietin receptor agonist (TPO-RA)

## Abstract

**Introduction:**

Immune checkpoint inhibitors (ICIs) are used in several advanced malignancies and may cause various immune-related adverse events (irAEs). Among them, hematological irAEs are less described. Acquired amegakaryocytic thrombocytopenia (AAT) is a rare immune hematologic disorder characterized by severe thrombocytopenia and complete absence of megakaryocytes in bone marrow.

**Case presentation:**

Herein, we present the case of a patient in their 40s with metastatic melanoma who developed an AAT after 12 cycles of nivolumab (anti-PD1). His platelet count decreased by ≤5 × 10^9^/l without other cytopenia. Bone marrow biopsy showed normal cellularity with a complete absence of megakaryocyte and T-CD8+ lymphocyte infiltration. Given the failure of systemic steroids, eltrombopag was started, an oral thrombopoietin receptor agonist (TPO-RA), and his platelet count subsequently increased with complete response.

**Discussion:**

Four other cases are described on literature with the same features than non-ICI-related AAT. All cases occurred after anti-PD/PD-L1 treatment with a median onset of 5 weeks. The presentation of our case is quite different with delayed cytopenia. Both ciclosporin and TPO-RA seem to be efficient therapies.

**Conclusion:**

TPO-RA could be preferred in oncologic patients, but safety data are still missing to define clear guidelines for immune-related AAT management.

## Introduction

Immune checkpoint inhibitors (ICIs) are now used in several advanced malignancies and may cause various immune-related adverse events (irAEs), including dermatological, endocrine (especially dysthyroidism and hypophysis), rheumatological, hepatic, or digestive (especially colitis) toxicities with incidences of >50%, 4%–20%, 10%, 5%–30%, and 1%–30%, respectively (1). Hematological irAEs (hem-irAEs) are quite rare with overall frequencies around 0.5% to 3.6% and 0.7% for grade ≥3 events (graded based on the National Cancer Institute Common Terminology Criteria for Adverse Events, version 5.0) (2, 3). Mortality of this hem-irAEs can be high especially for aplastic anemia and hemophagocytic lymphohistiocytosis (3). Immune thrombocytopenia (ITP) is the most frequent hematological AE, and anti-programmed death-1 (PD-1)/programmed death-ligand 1 (PD-L1) treatments are more frequently involved than anti-cytotoxic T-lymphocyte-associated protein 4 (CTLA-4) (2, 3).

Acquired amegakaryocytic thrombocytopenia (AAT) is a rare hematologic disorder characterized by severe thrombocytopenia and complete or nearly complete absence of megakaryocytes in the bone marrow (4, 5). Usually, AAT patients are poorly responders to standard ITP therapy (corticosteroids [CS] and intravenous immunoglobulin [IVIg]) (5). Only few cases of ICI-related AAT have so far been published, and given the rarity of this entity, no strong data on clinico-biological presentation and management are available (6–8). Herein, we describe another case of AAT after nivolumab perfusion and a literature review.

## Case presentation

**Figure 1:**
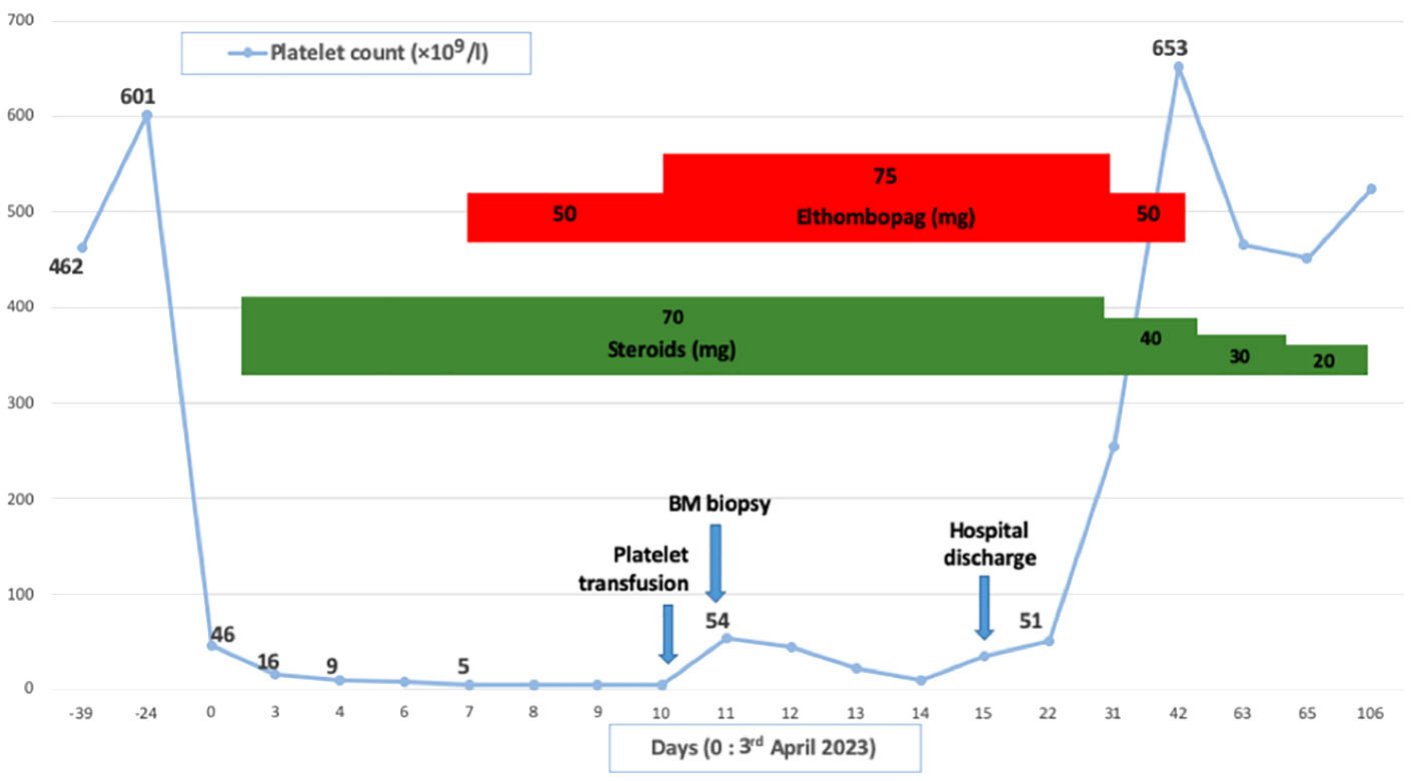
Clinical courses illustrating thrombocytopenia and main treatments. BM, Bone marrow.

**Figure 2:**
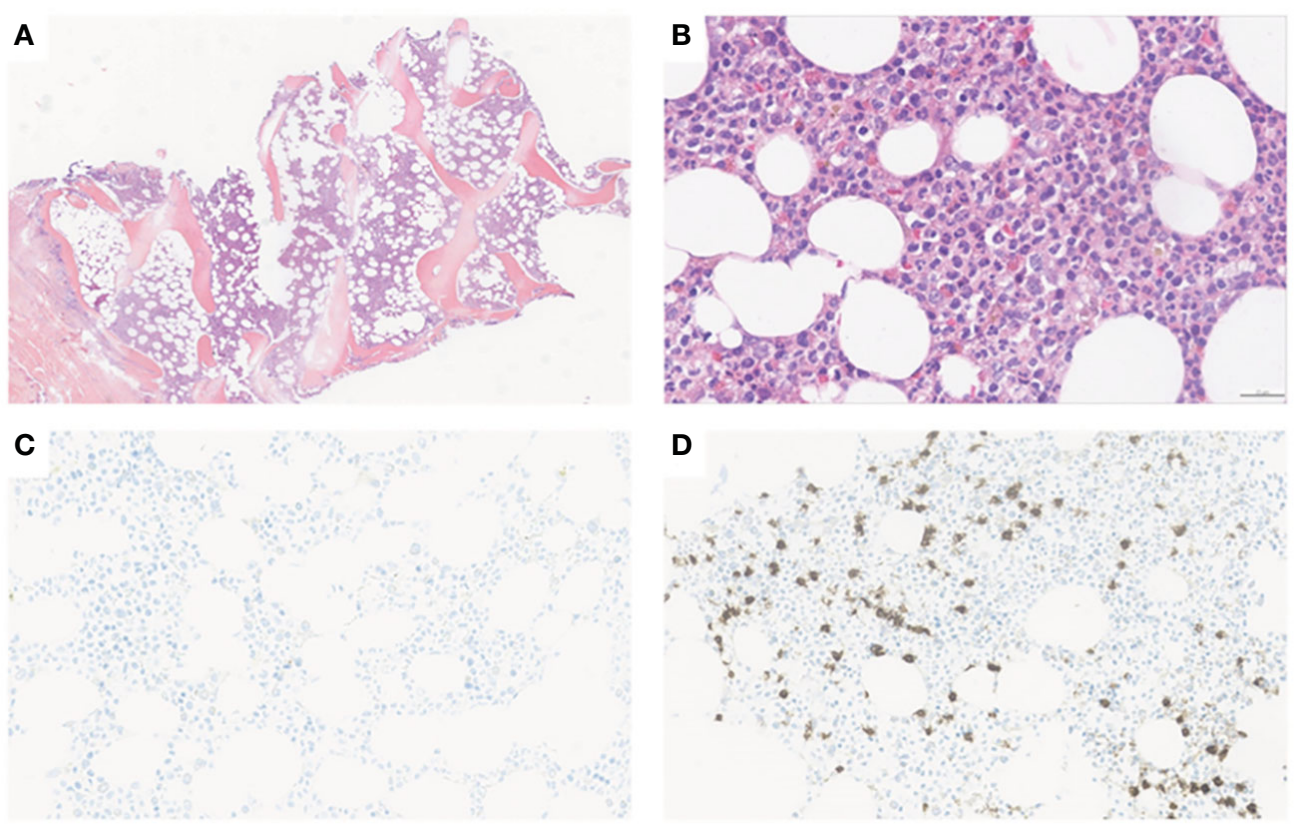
Bone marrow trephine biopsy. **(A)** Normocellular marrow (H&E, X5); **(B)** Absence of megacaryocytes and normal maturation pattern of erythroid and myeloid cells (H&E, X20). **(C)** Immunohistochemical staining with anti-CD61 confirms the absence of megacaryocytes (anti-CD61, x15). **(D)** Moderate neactive CD8+ T infiltrate (anti-CD8, x15).

Patient in their 40s with initially localized melanoma (Breslow 10 mm, *BRAF V600* positive) was treated by surgery. He had a personal history of Raynaud phenomenon and circulating anti-SSa, anti-SSb and anti-centromere antibodies. He had no hematological history. On August 2021, due to recurrence with metastatic involvement in the skin and lung, he started nivolumab perfusion (480-mg flat dose on a monthly basis). After four cycles, he suffered from a localized body-hair whitening (poliosis) within a tattoo on the upper chest. On January 2022, neoplastic assessment showed complete metabolic response and immunotherapy was continued. On January 2023, after 12 cycles (around 17 months), the blood sample revealed eosinophilia (1.3 G/l, N < 0.5 G/l) without any cytopenia, considered to be due to ICI and normalized after quick steroid therapy (1 mg per kilo or 60 mg per day during 1 week and then gradually tapering off over a period of 3 weeks). ICI was discontinued. On April, the patient suffered from severe thrombocytopenia and was referred to our hospital (**Figure 1**). He had no bleeding. The blood finding showed normal results except for platelet count ≤5 × 10^9^/l. No other organ disorder was suggested with not any evidence of disseminated intravascular coagulation or thrombotic microangiopathy in the laboratory data. A bone marrow (BM) smear and a BM biopsy showed normal cellularity with a complete absence of megakaryocytes associated with polyclonal T-CD8+ lymphocyte infiltration (negative clonal T-cell population assessment). There was no evidence of myelodysplastic neoplasia, myelofibrosis, or metastatic lesions (**Figure 2**). Cytogenetic analysis of BM showed a normal karyotype (46XY). Excluding other possible secondary diagnosis (other immune diseases, drugs, or infections), we concluded that AAT was caused by ICI. We started orally corticosteroid treatment (1 mg per kilo) on day 2, without any effect on platelet count. Platelet transfusion resulted in a moderate and transient rise in the platelet count. Finally, a treatment with eltrombopag [a thrombopoietin receptor agonist (TPO-RA)] was started on day 7 (oral 50 mg daily, increased to 75 mg after 4 days), by maintaining corticosteroids. In response, the platelet count subsequently improved after 7 days and the patient was hospital-discharged on day 15. Eltrombopag was tapered off and stopped (on day 42) after a platelet count of 653 G/l and then stabilized on a normal range. A new tumor assessment revealed a persistent complete metabolic response, and rechallenge of ICI had not yet been carried out.

## Discussion

AAT is an extremely rare cause of thrombocytopenia, but severe bleeding that is often life-threatening with a platelet count of ≤10 × 10^9^/l is a concern in most of the cases (5, 7). The underlying pathogenic mechanism of this disease is still unknown. An autoimmune mechanism and dysregulation of humoral immunity with antibodies against TPO (9) or c-MPL (thrombopoietin receptor) was considered. T-cell-mediated processes were also suggested and supported by the presence of T-cell CD8+ in BM biopsies and by *in vitro* inhibition of a donor or autologous megakaryocyte (MK) colony-forming unit (CFU) by T cells of AAT patients (5, 10, 11). Response to ciclosporin also suggested a T-cytotoxicity-mediated mechanism (5). Indeed, classic therapies such as CS and IVIg are only effective on 22.4% and 5.3% of AAT patients, respectively (5). Ciclosporin (CSA) seems more efficient with an overall response around 66% of patients (5). Other treatments such as anti-thymocyte globulin or rituximab have also been reported successful (7). TPO-RA, especially eltrombopag, is already used for ITP and aplastic anemia and seems to be also effective in AAT (5, 7). However, standard treatment guidelines are still missing in this disease.

Only four other cases of AAT after ICI are available in literature (**Table 1**) (6–8). Large series and pharmacovigilance database study about immune-related cytopenia did not include any cases (2, 3, 12). All AAT occurred after anti-PD/PD-L1 treatment and after very few cycles of immunotherapy with a median onset of 5 weeks. The presentation of our case is quite different with delayed hem-irAEs, which appears only after 12 cycles of nivolumab perfusion. Even when AAT is induced by ICI, thrombocytopenia is severe with platelet count ≤30 × 10^9^/l in all cases and ≤10 × 10^9^/l in three out of five (60%) patients. Other blood lines are classically preserved in AAT. Suyama et al. described a patient with only a moderate anemia (7). The second case published by Tu et al. also suffered from other cytopenia probably caused by secondary aplastic anemia (8). BM biopsy is important to exclude blast infiltration, dysplasia, aplastic anemia, or fibrosis. It revealed in all cases an absence of megakaryocytes. The cellularity of bone marrow is variable: clear hypoplasia was found in two patients (6, 8), moderate hypocellularity (20%–30%) in one patient, and normal one in two others, including our patient (8). In a surprising way, lymphocyte T-CD8+ infiltration, classically found in BM biopsy of an AAT patient and in other hem-irAEs such as aplastic anemia, is described only in our case (3, 5, 13). Platelet transfusion was tried in three cases with effect in only 2. In our case, the effect was very transient but allows BM biopsy without any bleeding complication that may suggest an associated peripheral mechanism. The various cases available in the literature are therefore heterogeneous in clinical presentation, in other cytopenia sometimes associated with thrombopenia, and in cellularity found in BM biopsy as well as unexpected response to platelet transfusion that suggested different physiopathological processes and not only typical AAT.

**Table 1:** Previously published and current case of immune checkpoint inhibitor-related acquired amegakaryocytic thrombocytopenia.

Author Ref (year)	Age/ Gender	Cancer	ICI	Onset*	Worst platelet count (×10^9^/l)	Grade CTCAE	Platelet transfusion	BM biopsy	Treatment/ Outcome of the irAEs/ Rechallenge
Rivet V, et al. (2024)	39/ Male	Melanoma	Nivo	96 (14)	<5	4	Effect	Normal cellularity marrow with absence of MK and T-CD8+ infiltration	CS + TPO-RA ICI discontinuation No rechallenge
Iyama S, et al. (5) (2020)	54/ Female	Pancreatic	Nivo	4 (2)	12	4	No effect	Hypocellular marrow with absence of MK	CS + IVIg and then CSA ICI discontinuation CR No rechallenge
Suyama, et al. (6) (2021)	78/ Male	Lung SCC	Durva	4 (2)	7	4	Effect	Moderately hypocellular marrow (20-30%) with absence of MK	CS and then TPO-RA ICI discontinuation CR No rechallenge
Tu, et al. (7) (2022)	56/ Male	Ureteric carcinoma	Tisle	6 (2)	21	4	Not used	Absence of MK with no significant abnormality	CS + IVIg and then CSA + TPO-RA CR No rechallenge
	71/ Male	Bladder carcinoma	Triple	6 (2)	2	4	Not used	Multilineage hypoplasia with near absence of MK	IVIg, CS and then TPO-RA ICI discontinuation CR No rechallenge

*Weeks (Number of perfusions) between initiation of immunotherapy and the diagnosis of the acquired megakaryocytes thrombocytopenia.

ICI, Immune Checkpoint Inhibitor; BM, Bone marrow examination; CTCAE, Common Terminology Criteria for Adverse Events; irAEs, immune-related Adverse Events; Nivo, Nivolumab; Durva, Durvalumab; Tisle, Tislelizumab; Triple, Triplezumab; CS, Corticosteroids; IVIg, Intravenous Immunoglobulin; TPO-RA, Thrompoietin Receptor Agonists; CSA, Ciclosporin; CR, Complete response; MK, megakaryocytes; SCC, Squamous Cell Carcinoma.

In all cases, ICI was discontinued (**Table 1**). According to data about non-ICI-related-AAT, CS and IVIg seem ineffective in all five AAT patients. In the case of Iyama et al., CS was nevertheless stopped after diagnosis of gastric ulcer and so response to steroid was not clear (6). CSA was used in two patients with complete response, defined as a platelet count ≥100×10^9^/l (14): alone in one case and associated with TPO-RA in another one (6, 8). In the three other cases, complete response was obtained after TPO-RA (7, 8). Both eltrombopag (50 mg daily) and avatrombopag has led to a quick increase of platelet count (7, 8). In our patient, both eltrombopag and CS were maintained. Because of the absence of risk of life-threatening bleeding, we did not prescribe IVIg. Platelet count finally increased after dose escalation of eltrombopag at 75 mg daily. However, the platelet count did not appear affected by introduction of this treatment at 50 mg daily. In this context, a delayed response to steroids or a spontaneous correction of thrombocytopenia cannot be ruled out and efficacy of eltrombopag is uncertain. In the end, standard first-line treatments such as CS and IVIg do not appear to be effective in this entity, and TPO-RA and CS should be considered. However, for now, data seem to be more significant for TPO agonists and the onset of action may be faster with these treatments. The optimal dose of eltrombopag in AAT patients is unknown. The usual dose in ITP patients of 50 mg–75 mg daily is currently used. However, higher doses, such as those used in aplastic anemia (150 mg–225 mg daily), could improve response to treatment but would require additional data.

## Conclusion

CSA and TPO-RA are both efficient in refractory AAT. In oncologic patients, TPO-RA could be promoted because of probably less risk of tumor progression than with CSA. Nevertheless, thrombotic risk with eltrombopag must be considered in this population (15). Finally, after immune-related cytopenia, rechallenge is rare with recurrence of the same hem-irAEs in more than 40% of patients (2). To our knowledge, no rechallenge was published in cases of AAT induced by ICI.

## Data availability statement

The original contributions presented in the study are included in the article/supplementary material. Further inquiries can be directed to the corresponding author.

## Ethics statement

Ethical approval was not required for the study involving human samples in accordance with the local legislation and institutional requirements. Written informed consent for participation in this study was provided by the participants’ legal guardians/next of kin. Written informed consent was obtained from the individual(s) for the publication of any potentially identifiable images or data included in this article.

## Author contributions

VR: Writing – original draft, Writing – review & editing, Conceptualization, Formal Analysis, Investigation, Validation. VS: Writing – review & editing. JD: Writing – review & editing. TV: Writing – review & editing. MB: Writing – review & editing. AP: Writing – review & editing. KD: Writing – review & editing. PC: Writing – review & editing. OR: Writing – review & editing. TC: Writing – original draft, Writing – review & editing.
